# The information needs of people living with ankylosing spondylitis: a questionnaire survey

**DOI:** 10.1186/1471-2474-13-243

**Published:** 2012-12-10

**Authors:** Roxanne Cooksey, Sinead Brophy, Muhammad Jami Husain, Elizabeth Irvine, Helen Davies, Stefan Siebert

**Affiliations:** 1 College of Medicine, Swansea University, Swansea, SA2 8PP, UK; 2 Keele Management School, Keele University, Keele, UK

**Keywords:** Ankylosing spondylitis, Information sources, Information needs, Education, Information usage

## Abstract

**Background:**

Today, health care is patient-centred with patients more involved in medical decision making and taking an active role in managing their disease. It is important that patients are appropriately informed about their condition and that their health care needs are met. We examine the information utilisation, sources and needs of people with Ankylosing Spondylitis (AS).

**Methods:**

Participants in an existing AS cohort study were asked to complete a postal or online questionnaire containing closed and open-ended questions, regarding their information access and needs. Participants were stratified by age and descriptive statistics were performed using STATA 11, while thematic analysis was performed on open-ended question narratives. Qualitative data was handled in Microsoft Access and explored for emerging themes and patterns of experiences.

**Results:**

Despite 73% of respondents having internet access, only 49% used the internet to access information regarding AS. Even then, this was only infrequently. Only 50% of respondents reported accessing written information about AS, which was obtained mainly in specialist clinics. Women were more likely than men to access information (63% (women) 46% (men)) regardless of the source, while younger patients were more likely to use online sources. The main source of non-written information was the rheumatologist. Overall, the respondents felt there was sufficient information available, but there was a perception that the tone was often too negative. The majority (95%) of people would like to receive a regular newsletter about AS, containing positive practical and local information. Suggestions were also made for more information about AS to be made available to non-specialist medical professionals and the general public.

**Conclusions:**

There appears to be sufficient information available for people with AS in the UK and this is mostly accessed by younger AS patients. Many patients, particularly men, choose not to access AS information and concerns were raised about its negative tone. Patients still rely on written and verbal information from their specialists. Future initiatives should focus on the delivery of more positive information, targeting younger participants in particular and increasing the awareness in the general population and wider non-specialist medical community.

## Background

Ankylosing Spondylitis (AS) is a chronic inflammatory arthritis that affects the axial skeleton. The disease typically occurs during the third decade of life [1-3] and affects 2.5 times more men than women [4]. The prevalence of ankylosing sponylitis is unknown although estimates from data from the UK and Hungary suggest a range between 0.05% to 0.23% [5].

Providing information to patients with chronic musculoskeletal diseases is recognised as good practice. There is, however, limited published literature exploring the information needs of people with arthritis [6-8], with the focus mainly on those with rheumatoid arthritis (RA) and osteoarthritis (OA). A study from Scandinavia found that patients with AS received significantly more information about diagnosis and medication than patients with RA [7]. There are also increasing numbers of websites providing information about AS, such as Patient.co.uk, National Ankylosing Spondylitis Society (NASS), Medicinenet.com, Arthritis Research UK and AS Assist [9-13].

As part of the MRC-funded Patient Research Cohort Initiative [14], we established the Population-based Ankylosing Spondylitis (PAS) cohort [15], which captures longitudinal data on patients with AS living in Wales, United Kingdom (UK). We wanted to provide the participants of the PAS study with information about AS and set out to examine the information needs and preferences of people with AS by exploring participant access, usage and opinion of available sources of AS information, an area which to date has been largely neglected.

## Methods

### Participants

People with AS who were resident in Wales, UK were recruited to the PAS cohort through their general practitioner (GP), rheumatologist, membership of the National Ankylosing Spondylitis Society (NASS), or through physiotherapy [15]. Information packs were sent directly to AS patients attending a rheumatologist inviting them to join the cohort; via participating GP surgeries whereby information packs were distributed from GP to AS patients; through regional NASS coordinators who sent the information packs to their members, and finally, from physiotherapists to their patients diagnosed with AS. This study draws on the data collected from members of the PAS cohort who completed the information needs questionnaire (please see additional 1 files for a copy of the questionnaire).

### Procedures

#### Questionnaire

All participants recruited as part of the PAS cohort were asked to complete (n = 418) a postal or online questionnaire about information needs, developed by a team of researchers and a rheumatologist at Swansea University. Participants were reminded by post or via email to complete the questionnaire, up to a total of two times in an attempt to improve participation rates.

The questionnaire consisted of open and close-ended questions to investigate the access and usage of information sources (e.g. electronic, paper based or health professionals), barriers and drivers for information use, frequency of information utilisation, appraisal of existing information and identification of information gaps. In addition, the Bath Ankylosing Spondylitis Disease Activity Index (BASDAI) [16], the Bath Ankylosing Spondylitis Functional Index (BASFI) [17] and a current health status measure, Euro-Qol-5 Dimensions (EQ-5D) [18] were included in the questionnaire. The BASADI consists of 10 cm visual analogue scales (VAS) used to answer 6 questions pertaining to the 5 major symptoms of AS. Scores range from 0–100 with scores of 40 or above suggesting suboptimal control of the disease. Similar to the BASDAI, the 10-item BASFI uses a 10 cm VAS to assess the AS patients’ functional ability. A score is calculated (0–100) to give an indication of patient disability. The EQ-5D comprises five dimensions: mobility, self-care, usual activities, pain/discomfort and anxiety/depression, and the respondent chooses an accompanying statement which indicates whether there are no problems, some problems or extreme problems in each of the dimensions. In addition, a 0–100 point VAS is used in the EQ-5D with the endpoints ‘Best imaginable health state’ (point 100 of scale) and ‘Worst imaginable health state’ (point 0 of scale).

#### Routine data

Working in collaboration with the Health Information Research Unit (HIRU) at Swansea University, we explored routinely collected GP records currently held on 1,592,484 patients in Wales, to examine whether response rate bias was an issue during this study. The HIRU’s Secure Anonymised Information Linkage (SAIL) databank [19], which currently holds over a billion records from various sources such as GP records, in-patient and out-patient data, overcomes confidentiality and disclosure issues by using a robust split-file approach [20].

Here, we utilised the GP records of the SAIL databank to explore the age and the gender of 1,920 people held in the database who were identified as having a diagnosis of AS and living in Wales, to compare with the demographic profile of the participants in our study.

#### Analysis of data

Participants were stratified by age (20–39 years, 40–59 years and over 60 years old). Descriptive statistics were calculated for participant characteristics using STATA 11. Scores for the questionnaire items were calculated as means and standard deviations.

The responses to open ended questionnaire items were handled in Microsoft Access and explored for patterned responses and emerging themes using thematic analysis.

Following a period of extensive data familiarisation which involved repeated and active reading, all open ended question responses were included for analysis and used to combine and catalogue a comprehensive picture of the collective experiences and opinions regarding the information needs of AS patients. A data-driven and semantic level approach to identifying was taken. Decided themes were coded accordingly by one researcher (RC) and refined in a recursive process as the data was analysed. The themes and coding practices were checked by another researcher (SB). A comprehensive account of thematic analysis is available elsewhere [21].

#### Ethical approval and consent

This study obtained ethical approval from the London Multi-centre Research Ethics Committee and the written consent of participants was obtained according to the declaration of Helsinki.

## Results

### Demographics

Questionnaires were completed by 211 out of 418 participants (50% response rate).

The sample was 81% male and the mean age of the respondents was 57 [see Table 1], with 10% (22/211) aged 20–39 years, 36% (77/211) aged 40–59 years and 52% (112/211) aged 60+. The sample was not representative of the whole PAS cohort as younger individuals participated less. 

**Table 1:** Respondent demographics

**Mean age (n = 211)**	**57 (S.D: 13)**
Male (n = 211)	81% (171/211)
Mean BASDAI (n = 211)	43 (S.D: 22)
Mean BASFI (n = 211)	51 (S.D: 28)
Age at diagnosis (n = 206)	33 (S.D: 12)
Age first symptoms (n = 203)	25 (S.D:10)
Disease duration from diagnosis (years) (n = 206)	23 (S.D: 14)
Disease duration from first symptom (years) (n = 203)	32 (S.D: 14)
Have children (n = 211)	73% (155/211)

In comparisons to Wales-wide data we found that Welsh resident patients diagnosed with AS, identified from GP records held in the SAIL databank (n = 1920), were 75% male, the mean age was 60, with 13% (241/1920) aged 20–39 years, 36% (683/1920) aged 40–59 years and 52% (996/1920) aged 60 and over. Demographic data is provided in Table 1.

### Information sources

#### Internet

The majority of respondents had access to the internet (73% (155/211); 95%CI: 67% to 79%)) with highest rates among younger participants (91% (20/22); 95%CI: 72% to 97%) aged 20–39.

The internet was used more for social networking by younger people than older people (64% (14/22); 95%CI: 43% to 80%) in those aged 20–39 compared to 7% (8/112); 95%CI: 3.6% to 13.5% in those aged 60 and over).

Less than half (49% (103/211); 95%CI: 42% to 56%) used the internet for information about AS, and of these, the frequency of online AS information searching was most commonly reported as “every 6 months or less” (58% (60/103); 95%CI: 49% to 67%).

The majority of younger participants (age 20–39) reported using the internet to find out about AS (77% (17/22); 95%CI: 56% to 90%) compared to 36% (40/112); 95%CI: 28% to 45%) of those aged 60 +.

The mean disease severity (BASDAI) and mean health status (EQ5D) for those who accessed online AS information (mean disease severity 43 (sd: 22) and mean health status 55 (sd: 22)) was similar to that of individuals who did not access online AS information (mean disease severity 43 (sd: 23) and mean health status 57 (sd: 23)). The main reasons given for not using the internet for AS information included not needing or wanting further information and not trusting information on the internet [Table 2]. 

**Table 2:** Quotes from comments made by respondents in the Information Needs Questionnaire

**Reasons not accessing AS information online:**	**Quote**
Don’t need further information	“I do not need to know any further information as I am already informed.” (Male, aged 70)
	“I think that there is enough information via leaflets, AS sites and health sites.” (Female, aged 43)
	“…I do not need to keep reading things. I know I have a stiff back but do not need to keep reading about things.” (Male, aged 51)
Don’t want further information	“Having AS is bad enough, I don’t want to read about it!” (Male, aged 62)
	“Don’t always feel like [using internet for AS information].” (Female, aged 50)
	“all doom and gloom”. (Male, aged 32)
Don’t trust the information	“It is difficult to know which sites to trust” (Male, aged 37)“
	“Possible safety implications [discourage me from accessing AS information online]”. (Female, aged 65)
Dislike Internet	“I don’t use the internet or a computer as I think it is the biggest backward set mankind has ever made.” (Male, aged 62)
	“A load of rubbish on it.” (Male, aged 64)
Time constraints	“I have an 18 month old that keep me busy – no time.” (Female, aged 38)
	“Can be time consuming – too much info.” (Male, aged 51)
Don’t think to look for information	“Don’t always think about looking as I have had AS for so long.” (Female, aged 50)
	“[Looking up AS online] Does not occur to me generally.” (Female, aged 71)
Prefer information from professional	“I prefer to talk to a professional, face to face.” (Male, aged 55)
	“I feel supported by my rheumatologist and have never felt the need to turn to anyone else…” (Female, aged 65)
Confusing information, do not understand	“Difficult to understand [information].” (Male, aged 63)
	“Not understanding properly what [information] I need.” (Female, aged 40)
**Suggestions for improving information and support:**	
Improved access to professionals/services	“More available access to AS professionals - I see physio once a year if I am lucky.” (Female, aged 44)
	“A more efficient means of accessing medical help. I have waited for up to 18 months to see a consultant.” (Male, aged 66)
	“The main issue is access to specialists. GPs often seem to know little about conditions such as AS and my consultants AS clinic (which is very good) only takes place every 3–6 months. There is a need to be able to discuss issues arising from flare-ups while they are occurring - not weeks or months later,” (Male, aged 37).
Happy with current level of AS Information	“I am happy now with the information I receive. However when I was really bad it was very hard to get to the people I needed to see.” (Female, 49)
	“Satisfied with the information and support that I have received.” (Female, 62)
	“As of the moment I am more than happy with the excellent level of support received from the rheumatology department at my local hospital - A veritable breath of fresh air, thank you.” (Male, 55)
Improved GP knowledge and support	“GP knows little about AS. GPs should be better informed and show interest at least.” (Male, aged 60)
	“Doctors that listen to you when you tell them that you get back pain so painful it gives me breathing problems.” (Male, aged 29)
	“I don’t always find GPs very knowledgeable about AS and sometimes they are quite dismissive of the condition.” (Female, aged 29)
AS groups or advice from others	“Swapping stories and self help, get AS sufferers to socialise with each other.” (Male, aged 34)
	“A helpline for people to contact for help e.g. for backup about the disease in relation to sickness claims and advice on drugs.” (Male, aged 47)
Information on cause and treatment	“Generally greater information on the cause of AS and the known treatments available. Plus what new treatments are coming onto the market or will be available in the near future.” (Male, aged 46)
More written information	“I would buy from book shops but I have never seen any books on AS.” (Male, aged 50)
	“I think the leaflets on AS should be displayed in GP surgeries or Hospitals. There are ones on Arthritis but nothing for AS.” (Male, aged 77)
More electronic information	“Better (more comprehensive) internet facility.” (Male, aged 41)
	“Regular emails to provide recent findings and other peoples experiences,” (Male, aged 36).
Updates to current news/research	“Updates regarding new treatments or therapies.” (Male, aged 66)
	“Regular feedback from health professionals as to research and different treatments available.” (Male, aged 46)
Better public awareness	“Not many people understand AS so if information was presented in simple language more people could have a better understanding of what people with AS suffer.” (Female, aged 61)
	“I think it would be a good idea to make people aware of AS through the media, perhaps then people would not shrug off an aching back for weeks on end before getting checked out…” (Male, aged 61)
	“More understanding from everyone, just even if people were more aware as with other inflammatory diseases.” (Male, aged 29)

The majority of respondents (79% (90/114); 95% CI: 71% to 85%)) rated the currently available online AS information as ‘helpful’. The information that people would like to see provided on the internet included summaries of the latest research and medications (61% (95/155); 95%CI: 53% to 69%)), the opportunity to ask a doctor questions (43% (66/155); 95%CI: 35% to 50%)) and AS networking (25% (39/155); 95%CI: 19% to 33%)).

From open-ended question responses, other information that people would like to see on the internet included help-lines, practical information about dealing with AS and a facility whereby patients could contact the rheumatology clinic they attend, although the numbers reporting these were small.

#### Written information

Only half of all the respondents reported obtaining written information on AS (50% (105/211), 95%CI: 43% to 56%). This was obtained mostly from hospital clinics (33% (70/211), 95%CI: 27% to 40%) national charities (18% (39/211); 14% to 24%)) and GP surgeries (12% (25/211); 95%CI: 82% to 17%) but rarely from libraries (3% (7/211); 95%CI: 2% to 7%), and books or other places (2% (5/211); 1% to 5%). For those who had no access to the internet, 61% (34/56; 95%CI: 48% to 72%) picked up written information compared to 46% (71/155; 95% CI: 38% to 54%) with internet access.

The vast majority of patients (95%, (157/166)) reported that they would like to receive a regular or occasional newsletter about AS. The majority of participants stated that they would like to see summaries of the latest AS research (65% (138/211); 95%CI: 59% to 71%), stories and experiences from other AS patients (43% (90/211; 95%CI: 36% to 49%), an opportunity to ask a doctor about questions (35% (74/211; 95%CI: 29% to 42%) and information about local events (27% (56/211); 95%CI: 21% to 33%) in newsletters about AS.

From open-ended question responses provided, participants appear to favour practical information, medication information and self-help guidance over purely factual information about the disease course itself and would like to see such information contained in AS newsletters. Participants felt that information on the disease itself was readily available and reading it would not change their outcome and as such, was of limited help to them. Some even replied that they would prefer to not know about this information; “It never has a positive angle, it’s always ‘this will or could happen’, [*it’s all*] doom and gloom” [Table 2].

#### Non-written information sources

The rheumatologist (56% (117/209); 95%CI: 49% to 63%), the GP (37% (77/209); 95%CI: 31% to 44%) and the nursing and physiotherapy team (35% (68/209); 95%CI: 27% to 39%) were the main sources of non-written or non-electronic information. Friends and family were used the least as a source of information (20% (41/209; 95%CI: 15% to 26%).

### Information needs by age and gender

Rates of AS information gathering were higher in females than in males, regardless of the source of information, 66% (27/41) of females accessed online AS information compared with 46% (79/170) of males (difference: 19%; 95%CI: 2.3% to 34%) and similarly, 63% (26/41) of females obtained written AS information compared with 46% (79/170) of males (difference 17%; 95%CI: 0% to 32%).

Younger participants aged 20–39 years appear to have some differing information needs from older participants; younger participants had higher rates for accessing AS information online (77%, 17/22) and lower rates for obtaining written information (41%, 9/22) (difference 36%; 95%CI: 7% to 58%).

Thematic analyses revealed that older participants more frequently reported that they were happy with the level of AS information available and that they did not want any more information. All age groups expressed the view that GP knowledge and support, access to specialist healthcare professionals and services, AS groups or advice from others and updates of current news and research could be improved [Table 2].

### Improving information delivery

When asked in an open question about how AS information and support could be improved, the most commonly reported recommendation was for improved access to AS specialists and services. Many participants stated that they were happy with the current level of AS information available to them. However, participants would like to see improved GP knowledge and support and improved public awareness about AS [Table 2].

## Discussion

People with AS in our cohort appear to be largely content with the level of information currently available to them and feel generally well informed about their condition which replicates previous findings in people with AS [7,21]. This is not apparent for individuals with RA where a lack of knowledge has been reported [7,22-24]. Furthermore, we found that many participants did not want any further information about AS. This could reflect the higher proportion of male patients with AS compared to RA. Indeed, here we found that in all age groups, women were more likely to seek information about AS than men.

The majority of our respondents were older (mean age 57 years), and would therefore have had AS for several decades. AS is generally a slow, progressive disorder, so many patients with long-standing disease would have learned to cope and manage their condition. In contrast, the younger participants did require further information and the majority of this group used the internet for this purpose. Therefore, the information needs for newly diagnosed AS patients appear to differ from those with established disease and information provision should be targeted to younger patients.

Several respondents perceived available information about AS as “doom and gloom” and many respondents expressed that they would rather not know about all of the complications and problems associated with the condition. As such, it appears that the tone of the information is too negative and may explain why participants generally reported they were ‘satisfied’ with the amount of AS information available, when in fact they did not want to read any more depressing information. In RA, patients have described the need for additional information regarding therapies and treatments, scientific developments and social support, improving symptoms and quality of life while information about the disease itself was mentioned last [24]. This suggests that information on new therapies and proactive advice is more useful to patients with chronic inflammatory arthritis, rather than information relating to the mechanisms, complications and prognosis of the disease itself, which does little to help improve quality of life or health outcomes. New information should aim to be more positive to avoid making people with AS feel despondent when reading about their condition, particularly now that there are effective therapies available for AS.

The utilisation rates of written AS information and online information were quite low (50% and 49%, respectively) and accessed infrequently (on average every 6 months or less), with older participants most frequently accessing written sources and the younger accessing predominantly online information. Previous studies have shown that new rheumatology patients look up their symptoms prior to their first rheumatology appointment and that 62.5% of patients sought this information online [25].

Despite participants reporting low utilisation rates of both written and electronic information, and frequently stating they neither wanted nor needed further information, the vast majority would still like to receive a newsletter. This suggests that AS patients are interested in regular updates of new and relevant AS information in a newsletter format. This could be achieved at a relatively low cost and involve people with AS at the design stage to ensure that the content and tone is appropriate.

Participants wanted easier access to specialists for information, suggesting a need for more personalised information rather than generic information about AS, which is a heterogeneous condition with a variable course. However, we realise that it is not necessarily practical for AS patients to enjoy easier access to specialists, due to availability and costs associated with specialised, one-to-one healthcare on a more regular basis. Further investigations may be useful to explore the costs related to more regular contact with a specialist compared with costs associated with increased visits to the GP and utilisation of other and perhaps less appropriate health care provisions that may occur as a result.

Rather than wanting more information for themselves, many respondents felt there was a need for improved information for non-specialist healthcare professionals and the general public. Again, this may reflect the fact that the majority of our respondents were older with longstanding disease, and therefore had an understanding of their own disease, but still felt that others did not understand it. This could be a particular issue for AS where the main burden of disease is in the spine with relatively little to see in terms of peripheral joint swelling or destruction compared to conditions like RA or OA. AS is associated with fatigue and unpredictable flares of disease activity which may be difficult for others to appreciate and understand. With this in mind, future initiatives could be developed to help raise the awareness of AS for GPs and primary care teams in order to extend the available support for AS patients which may also help address the delay in AS diagnosis. The participation of non-specialist medical professionals in local groups and training sessions may serve to help address this.

As is the case in this study, the need for GPs to be better informed has also been highlighted in a study with multiple sclerosis patients [26] and similarly in a focus group study with elderly participants, where lack of time with GPs and questions left unanswered by health care providers was a barrier to participants understanding health information [27]. Information from GPs and primary care teams will become increasingly important as the long-term management of chronic conditions is increasingly being delivered in the community and primary care settings, with limited access to specialist services.

In addition to improved GP awareness, AS patients also feel that greater public awareness of AS is required and so projects to help raise the profile of AS are likely to benefit the AS community. The College of Medicine at Swansea University have organised successful AS Awareness days and events that have attracted media attention, which we feel helps raise awareness of the condition.

### Limitations of the study

The response rate for this questionnaire was relatively low (50%), compared to the response rates of the same cohort of AS patients for other questionnaires about disease flares, fatigue or work which have all been ≥70%. This low rate may suggest ambivalence regarding the issue of AS information which is reflected in the open question responses. Indeed, similar low responses have been reported in other studies investigating the information needs of patients with arthritis (58% response rate) [7,22] and 63% [23].

The majority of younger patients in the PAS cohort did not reply to the questionnaire. Therefore, the findings of the study can only reflect the responses of participants who are motivated to complete this questionnaire and this group may have different views from those who did not participate. The majority of respondents were older, and by definition would have longstanding disease and their information needs may differ significantly from patients with a recent diagnosis of AS. For example, younger men may not be receptive to information in the early stages and this group of young men may be difficult to reach effectively using traditional means. However, this study was stratified by age in order to compare the information needs between the different age groups.

This study has revealed a general lack of need for more patient information about AS and a discontentment with the negative content of current available information, which may explain the apparent apathy in completing a questionnaire about AS information.

## Conclusions

Patients with AS are generally satisfied with the amount of information available to them, but not the negative tone in which it is delivered. Information provided ought to be more practical and positive, particularly now that effective therapies are available for AS. Information utilisation and needs differ across gender and age groups, which should be considered in order to reach all target populations. The internet is a useful tool for delivering AS information to those who seek it; predominantly the youngest generation and websites and forums allow the sharing of experiences and offer practical and encouraging advice.

The importance of specialist rheumatology services as sources of information was identified by participants, as well as the need for improved non-specialist healthcare professional and public awareness of AS. Healthcare professionals with expertise in AS, in conjunction with AS patients should be involved in developing strategies to address this information gap.

## Abbreviations

AS: Ankylosing spondylitis; BASDAI: Bath ankylosing spondylitis disease activity index, BASDAI; CI: Confidence intervals; BASFI: Bath ankylosing spondylitis functional index; EQ-5D: Euro-Qol 5 dimensions; GP: General practitioner; NASS: National ankylosing spondylitis society; OA: Osteoarthritis; PAS: Population-based ankylosing spondylitis (cohort); RA: Rheumatoid arthritis.

## Competing interests

Authors confirm that there are no competing interests.

## Authors’ contributions

RC: made substantial contributions to conception and design of the study, acquisition of data, analysis and interpretation of data, drafting and revising of manuscript. SB: substantially contributed to conception and design of the study, acquisition of data, analysis and interpretation of data, drafting and revising of manuscript. MJH: carried out analysis and interpretation of data and was greatly involved in drafting and revision of the manuscript. EI: made substantial contributions to conception and design of the study, acquisition of data and drafting and revision of the manuscript. HD: substantially aided in analysis and interpretation of results and drafting and revision of the manuscript. SS: substantially contributed to conception and design of the study, acquisition of data, analysis and interpretation of data, drafting and revising of manuscript. All authors have read and approved the final manuscript.

## Pre-publication history

The pre-publication history for this paper can be accessed here:

http://www.biomedcentral.com/1471-2474/13/243/prepub

## Supplementary Material

Additional file 1 Information questionnaire completed by participants. Click here for file
