# Supplementary material for: The information needs of people living with ankylosing spondylitis: a questionnaire survey
Source: BMC Musculoskelet Disord. 2012 Dec 10;13:243. doi: 10.1186/1471-2474-13-243 (PMC3553011; doi:10.1186/1471-2474-13-243)
Supplement: Additional file 1 — Information questionnaire completed by participants. [file 1471-2474-13-243-S1.doc]

**Table 2: Quotes from comments made by respondents in the Information Needs Questionnaire**

|  |  |
| --- | --- |
| **Reasons not accessing AS information online:** | **Quote** |
| Don’t need further information | “I do not need to know any further information as I am already informed.” (Male, aged 70)  “I think that there is enough information via leaflets, AS sites and health sites.” (Female, aged 43)  “…I do not need to keep reading things. I know I have a stiff back but do not need to keep reading about things.” (Male, aged 51) |
| Don’t want further information | “Having AS is bad enough, I don’t want to read about it!” (Male, aged 62)  “Don’t always feel like [using internet for AS information].” (Female, aged 50)  “all doom and gloom”. (Male, aged 32) |
| Don’t trust the information | “It is difficult to know which sites to trust” (Male, aged 37)  “Possible safety implications [discourage me from accessing AS information online]”. (Female, aged 65) |
| Dislike Internet | “I don’t use the internet or a computer as I think it is the biggest backward set mankind has ever made.” (Male, aged 62)  “A load of rubbish on it.” (Male, aged 64) |
| Time constraints | “I have an 18 month old that keep me busy – no time.” (Female, aged 38)  “Can be time consuming – too much info.” (Male, aged 51) |
| Don’t think to look for information | “Don’t always think about looking as I have had AS for so long.” (Female, aged 50)  “[Looking up AS online] Does not occur to me generally.” (Female, aged 71) |
| Prefer information from professional | “I prefer to talk to a professional, face to face.” (Male, aged 55)  “I feel supported by my rheumatologist and have never felt the need to turn to anyone else…” (Female, aged 65) |
| Confusing information, do not understand | “Difficult to understand [information].” (Male, aged 63)  “Not understanding properly what [information] I need.” (Female, aged 40) |
| **Suggestions for improving information and support:** |  |
| Improved access to professionals/services | “More available access to AS professionals - I see physio once a year if I am lucky.” (Female, aged 44)  “A more efficient means of accessing medical help. I have waited for up to 18 months to see a consultant.” (Male, aged 66)  “The main issue is access to specialists. GPs often seem to know little about conditions such as AS and my consultants AS clinic (which is very good) only takes place every 3 - 6 months. There is a need to be able to discuss issues arising from flare-ups while they are occurring - not weeks or months later,” (Male, aged 37). |
| Happy with current level of AS Information | “I am happy now with the information I receive. However when I was really bad it was very hard to get to the people I needed to see.” (Female, 49)  “Satisfied with the information and support that I have received.” (Female, 62)  “As of the moment I am more than happy with the excellent level of support received from the rheumatology department at my local hospital - A veritable breath of fresh air, thank you.”(Male, 55) |
| Improved GP knowledge and support | “GP knows little about AS. GPs should be better informed and show interest at least.” (Male, aged 60)  “Doctors that listen to you when you tell them that you get back pain so painful it gives me breathing problems,” (Male, aged 29).  “I don’t always find GPs very knowledgeable about AS and sometimes they are quite dismissive of the condition.” (Female, aged 29) |
| AS groups or advice from others | “Swapping stories and self help, get AS sufferers to socialise with each other.” (Male, aged 34)  “A helpline for people to contact for help e.g. for backup about the disease in relation to sickness claims and advice on drugs.” (Male, aged 47) |
| Information on cause and treatment | “Generally greater information on the cause of AS and the known treatments available. Plus what new treatments are coming onto the market or will be available in the near future.” (Male, aged 46) |
| More written information | “I would buy from book shops but I have never seen any books on AS.” (Male, aged 50)  “I think the leaflets on AS should be displayed in GP surgeries or Hospitals. There are ones on Arthritis but nothing for AS.” (Male, aged 77) |
| More electronic information | “Better (more comprehensive) internet facility.” (Male, aged 41)  “Regular emails to provide recent findings and other peoples experiences,” (Male, aged 36). |
| Updates to current news/research | “Updates regarding new treatments or therapies.” (Male, aged 66)  “Regular feedback from health professionals as to research and different treatments available.” (Male, aged 46) |
| Better public awareness | “Not many people understand AS so if information was presented in simple language more people could have a better understanding of what people with AS suffer.” (Female, aged 61)  “I think it would be a good idea to make people aware of AS through the media, perhaps then people would not shrug off an aching back for weeks on end before getting checked out…” (Male, aged 61)  “More understanding from everyone, just even if people were more aware as with other inflammatory diseases” (Male, aged 29) |
